# [Corrigendum] Induction of cardiomyocyte-like cells from hair follicle cells in mice

**DOI:** 10.3892/ijmm.2026.5857

**Published:** 2026-05-13

**Authors:** Yong-Hee Kim, Bang-Jin Kim, Seok-Man Kim, Sun-Uk Kim, Buom-Yong Ryu

Int J Mol Med 43: 2230-2240, 2019; DOI: 10.3892/ijmm.2019.4133

Following the publication of the above article, an interested reader drew to the authors' attention that, concerning the micrographic images shown in Fig. 3B on p. 2234, the 'OP9' and 'MEF' data panels showed an overlapping section, such that data which were intended to show the results from differently performed experiments had apparently been derived from the same original source.

Upon investigating this figure, the authors realized that, during the revision process when several figures needed to be replaced with higher-quality images, an inadvertent error occurred, resulting in the unintentional duplication of the 'MEF' cell image in the 'OP9' data panel. The revised version of Fig. 3, now showing the correct data for the 'OP9' panel, is shown on the next page. The authors confirm that the error made when this figure was reassembled did not have a significant impact on either the results or the conclusions reported in this study, and all the authors agree with the publication of this Corrigendum. The authors are grateful to the Editor of *International Journal of Molecular Medicine* for allowing them the opportunity to publish this Corrigendum; furthermore, they apologize to the readership of the Journal for any inconvenience caused.

## Figures and Tables

**Figure 3:**
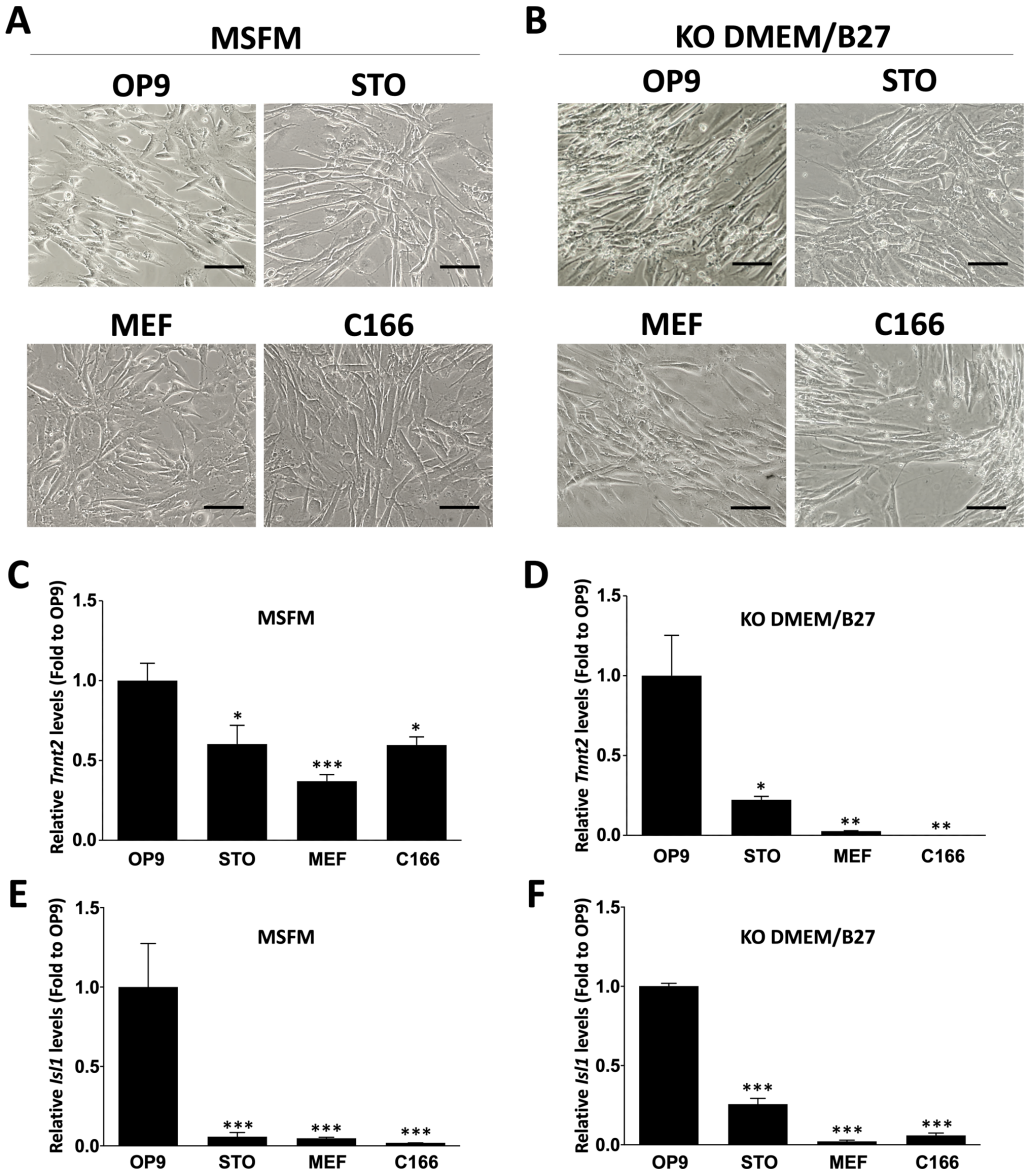
Cardiac differentiation of HF cells on feeder layers. Bright field micrographs of HF cells cultured on OP9, STO, MEF and C166 feeder cells in (A) MSFM or (B) KO-DMEM/B27 medium containing VEGF. Bar, 100 μm. Relative mRNA expression levels of Tnnt2 in (C) MSFM and (D) KO-DMEM/B27 groups. Relative expression of Isl1 in (E) MSFM and (F) KO-DMEM/B27 groups. Expression levels were normalized compared with those in the OP9 feeder cell group. Data is represented as the mean ± the standard error of the mean (n=3). ^*^P<0.05, ^**^P<0.01 and ^***^P<0.001 vs. the OP9 group. HF, hair follicles; MSFM, mouse serum-free medium; KO-DMEM, KnockOut-Dulbecco's modified Eagle's medium; Tnnt2, troponin T2, cardiac type 2; Isl1, islet 1; VEGF, vascular endothelial growth factor.

